# Modeling of Antioxidant Activity, Polyphenols and Macronutrients Content of Bee Pollen Applying Solid-State ^13^C NMR Spectra

**DOI:** 10.3390/antiox10071123

**Published:** 2021-07-14

**Authors:** Sylwester Mazurek, Roman Szostak, Mateusz Kondratowicz, Magdalena Węglińska, Agnieszka Kita, Agnieszka Nemś

**Affiliations:** 1 Department of Chemistry, University of Wrocław, 14 F. Joliot-Curie, 50-383 Wrocław, Poland; mateusz.kondratowicz@chem.uni.wroc.pl (M.K.); magdalena.weglinska@chem.uni.wroc.pl (M.W.); 2 Department of Food Storage and Technology, Faculty of Biotechnology and Food Science, Wrocław University of Environmental and Life Sciences, 37 Chełmońskiego, 51-630 Wrocław, Poland; agnieszka.kita@upwr.edu.pl (A.K.); agnieszka.nems@upwr.edu.pl (A.N.)

**Keywords:** bee pollen, ^13^C NMR spectroscopy, chemometrics, nutrients, polyphenols, antioxidant activity, quantitative analysis

## Abstract

An application of solid ^13^C nuclear magnetic resonance (NMR) spectroscopy for the determination of macronutrients, total polyphenols content, antioxidant activity, N C S elements, and pH in commercially available bee pollens is reported herein. Solid-state ^13^C NMR spectra were recorded for homogenized pollen granules without chemical treatment or dissolution of samples. By combining spectral data with the results of reference analyses, partial least squares models were constructed and validated separately for each of the studied parameters. To characterize and compare the models’ quality, the relative standard errors of prediction (RSEP) were calculated for calibration and validation sets. In the case of the analysis of protein, fat and reducing sugars, these errors were in the 1.8–2.5% range. Modeling the elemental composition of bee pollen on the basis of ^13^C NMR spectra resulted in RSEP_cal_/RSEP_val_ values of 0.3/0.6% for the sum of NHCS elements, 0.3/0.4% for C, 1.8/1.9% for N, and 4.2/6.1% for S quantification. Analyses of total phenolics and ABTS antioxidant activity resulted in RSEP values in the 2.7–3.5% and 2.8–3.8% ranges, respectively, whereas they were 1.4–2.1% for pH. The obtained results demonstrate the usefulness of ^13^C solid-state NMR spectroscopy for direct determination of various important physiochemical parameters of bee pollen.

## 1. Introduction

Bee pollen, which contains almost all nutrients necessary to the human diet, is one of the oldest nutritional supplements. It is composed of flower pollen, i.e., units of male gametophytes of flowering plants, mixed with nectar and bee salivary secretions. Bee pollen is used by worker bees for bee bread production, which provides the basic food for the larval queen and worker larvae [1].

The color of pollen grains ranges from white or creamy white to yellow and orange, as well as red, green, gray, violet, and dark brown, depending on the plant species from which it originates. Physiochemical, functional, and sensory characteristics are usually relatively fixed for mono-floral pollens of a particular botanical origin, whereas multi-floral pollen loads vary in their properties. In the case of the same plant source, pollen composition may change due to geographical origin, climate, weather conditions, soil type, or even the time of harvesting and the breed of bees [2]. About 250 biologically active substances have been identified in bee pollen grains. They contain carbohydrates (13–55%), protein (10–40%), lipids (1–13%), crude fiber (0.3–20%), and mineral constituents (ash content 2–6%), and they include all essential amino acids and a number of fatty acids, vitamins, carotenoids, and flavonoids. Fructose is the major sugar present in bee pollen, followed by glucose and sucrose. Arabinose, isomaltose, melibiose, melezitose, ribose, trehalose, and turanose account for nearly 1% of remaining sugars in pollen [3,4].

The biological activity of bee pollen is strongly related to its relatively high polyphenolic compound content, which is mainly responsible for its powerful antioxidative effects [5]. Among the antioxidants present in bee pollen, low molecular weight compounds are the most significant. Ascorbic acid and polyphenolic compounds are hydrophilic, whereas tocopherols and carotenoids are hydrophobic antioxidants. Water-soluble antioxidants, including vitamin C, scavenge hydroxyl radicals [6]. The concentration of these bioactive compounds in bee pollen, like its nutritional composition, depends on the source of its origin [1,7,8].

Nuclear magnetic resonance (NMR) spectroscopy is one of the most powerful and versatile analytical techniques that can be applied to the study of liquid and solid materials. It is a robust method that enables rapid analysis of mixtures at the molecular level without the need for separation or purification steps. In food science, the ^1^H NMR technique has an established position as an important analytical tool [9,10,11,12].

^1^H and ^13^C NMR spectroscopies were applied to differentiate the botanical origins of honeys, authenticate them, and detect adulteration [13,14,15,16]. The ^13^C NMR spectra were used to quantify saccharides in Greek honey samples [17] and in a quantitative analysis of carbohydrates in multi-component laboratory prepared mixtures [18]. Solid-state NMR spectra were applied to observe temperature-dependent changes in the molecular structures of honeybee wax and silk [19]. In bee pollen studies, the ^13^C NMR technique has been applied in the analysis of the composition of bee pollen color fractions [20].

An important issue related to the application of modern spectroscopic methods to the analysis of multicomponent systems is the dimensionality and complexity of the recorded data [21]. The use of multivariate data analysis techniques allows for the qualitative and quantitative analysis of the studied systems, even in the case of strongly overlapped signals or highly similar spectra. The decomposition of spectral matrices using chemometric techniques, e.g., principal component analysis (PCA) or partial least squares (PLS) regression algorithms, facilitates the filtering of the signal of interest even from the noisy spectra, allowing for the analysis of variance related to structural changes of the system. The use of chemometrics supports classification and quantification based on the NMR spectra [22,23,24], which establishes application in metabolomics, and the analysis of natural products and complex biological medicines [25,26].

In this report, the results of bee pollen analysis obtained by applying solid-state NMR spectroscopy are presented. Although ^13^C NMR spectra are considered less useful in the analysis of complex multicomponent systems compared to ^1^H NMR spectra because of their noticeably lower sensitivity, we demonstrate their utility in the multivariate modeling of selected parameters of such a complicated system. The spectra of powdered pollen granules were used to build PLS models that facilitate reliable quantification of macronutrients and determination of selected other parameters of the studied bee pollens, including total polyphenols content, ABTS antioxidant activity, and pH.

## 2. Materials and Methods

### 2.1. Experimental Material

Thirty samples of bee pollen collected from Polish beekeeping farms during the 2018 and 2019 seasons were mixtures composed of several types of granules varying in color from pale yellow to almost black. An additional five samples were prepared by manual selection of similarly colored granules. Each sample was powdered in a mill and divided into parts for NMR and reference analyses; details of the analyzed samples are summarized in Table 1.

### 2.2. Reference Analyzes

To prepare extracts, portions of the powdered pollen (0.1 g) were dissolved in 5 mL of methanol–water solution (70%, *v*/*v*). Samples were shaken for 10 min in a Vortex mixer, ultrasonicated and centrifuged (10,000 rpm for 10 min). The obtained extracts were decanted and stored for analysis.

Quantification of reducing sugars, after hydrolysis, was performed according to the AOAC 968.28 procedure [27]. Nitrogen content was evaluated according to the AOAC 981.10 protocol based on the Kjeldahl method [28], and protein concentration was calculated using a conversion factor of 6.25. Fat in the pollen samples was determined with the AOAC 963.15 Soxhlet method [29], using a B-811 universal extraction system (Büchi, Flawil, Switzerland).

Total polyphenols (TP) content was determined by following the Folin–Ciocalteu method, using a UV-2401PC spectrometer (Shimadzu, Kyoto, Japan). The system was standardized for gallic acid (GAE) in the 0–500 μg/mL concentration range. The obtained calibration curve for GAE was characterized by the determination coefficient (R^2^) value of 0.998. The antioxidant activity was measured using the colorimetric determination of the 2,2′-azino-bis(3-ethylbenzothiazoline-6-sulphonic acid) (ABTS) radical cation formed in the presence of potassium persulfate [30].

Pollen sample pH was measured in an aqueous phase by applying a S220 pH meter (Mettler Toledo, Greifensee, Switzerland) and CHNS elemental analysis was performed utilizing Vario EL Cube analyzer (Elementar Analysensysteme, Langenselbold, Germany) equipped with a thermal conductivity detector.

### 2.3. Chemicals and Reagents

Methanol, Folin–Ciocalteu reagent, ABTS, sodium hydrogen sulfite (NaHSO_3_), and formic acid were purchased from Sigma-Aldrich (Steinheim, Germany). 3,5-dinitrosalicylic acid (DNS) used for reducing sugars determination was purchased from Carbosynth (Bershire, UK) and diethyl ether originated from Chempur (Piekary Śląskie, Poland). All chemicals used in the experiment were of analytical grade.

### 2.4. Measurement Conditions

Cross-polarization magic-angle spinning NMR spectra were recorded on a Bruker Avance III 300 MHz (Bruker Biospin, Ettlingen, Germany) spectrometer equipped with 4-mm broad band CP-MAS probe head. Approximately 100 mg of the studied material in the form of powder was placed into a 4-mm zirconium oxide rotor. Using a magic angle spinning rate of 10 kHz, ^13^C NMR spectra were acquired with 3072 data points and 5120 scans with a 5 s recovery delay; they were processed using TopSpin (v3.62, Bruker Biospin, Ettlingen, Germany) software. The chemical shifts were referenced to adamantane as an external standard.

### 2.5. Multivariate Modeling

A typical quantitative analysis applying NMR spectra involves finding a ratio between the intensity of a peak of an internal standard, added in a known amount to the analyzed mixture, and the intensity of a band in a spectrum originating from a particular compound. A univariate approach based on intensity facilitates the determination of a compounds’ absolute content. This procedure requires an appropriate chemical substance that can serve as an internal standard, contains the nucleus of interest, and has resonances, for which the signal contributions of the analyte do not overlap and are chemically inert. Without adding the standard, only the relative concentrations of components can be determined. It may be difficult to find an appropriate standard, especially in the case of the analysis of complex solid samples and the simultaneous quantification of a number of components.

There is, however, another quantitative analysis method that does not require an internal standard. Suitable calibration models can be constructed based on the spectra of samples with known compound concentrations. Once the relation between variability in the spectral data and the chemical composition is established, the model can predict parameters of unknown samples. The most popular algorithm used in such a quantitative analysis is PLS regression, which belongs to the supervised calibration method group. The purpose of PLS is to construct a linear regression model that enables the prediction of a desired feature from a measured multivariate signal. In PLS, the data matrix X, here a spectral matrix, is resolved into components, or so-called latent variables. The general model can be defined as follows:

Y = X B + E(1)

where Y is matrix of reference data, X contains spectral data, B represents a matrix of regression coefficients, and E is a matrix of residuals not explained by the model. During model construction, the X and Y matrices are decomposed into scores and loadings, according to the following equations:

X = T P^T^ + E_X_ (2)

Y = U Q^T^ + E_Y_, (3)

where T and U are score matrices and P and Q are loadings of X and Y, respectively. PLS is a two-block regression method, so the decomposition of X is performed as a function of Y in a simultaneous analysis of the two data sets. Therefore, the PLS algorithm actively reduces the influence of X variation not correlated to Y [24].

### 2.6. Software and Numerical Data Treatment

Principal component analysis (PCA) of spectral data was performed in the Matlab environment (v7.10, MathWorks, Natic, MA, USA) applying PLS-Toolbox (v6.2, Eigenvector Research, Manson, WA, USA). Regression models were built utilizing TQ Analyst software (v9, Thermo Fisher Scientific, Waltham, MA, USA).

For PCA and PLS modeling purposes, spectral data were mean-centered. When necessary, additional normalization applying multiplicative scatter correction (MSC) or standard normal variate (SNV) procedures was performed. Combining the bootstrap method and the score plots of PCA, twenty-five samples were selected for calibration and ten others were selected for an external validation procedure. A cross-validation root mean square error was calculated to establish an optimal number of factors for the PLS modeling. To compare the predictive abilities of the constructed models, the relative standard errors of prediction (RSEPs) for calibration and validation sets were calculated according to the following equation:

(4) $$RSEP\left(\%\right)=\sqrt{\frac{\sum_{i=1}^{n}{\left(C_{i}-C_{i}^{A}\right)}^{2}}{\sum_{i=1}^{n}C_{i}^{A}{}^{2}}}\times 100,$$

where *C^A^* is a parameter value determined by the reference method, *C* is the value found from PLS modeling, and *n* is the number of samples. This measure is closely related to the commonly used root mean square error (RMSE):

(5) $$RSEP\left(\%\right)=\sqrt{\frac{n}{\sum_{i=1}^{n}C_{i}^{A}{}^{2}}}RMSE\times 100,$$

however, it does not depend on the units in which the measured parameters are expressed. An internal validation of the elaborated models was performed using the leave-one-out procedure, while an external validation involved the determination of the *RSEP_val_* errors for validation samples not included during the construction of the respective models.

## 3. Results and Discussion

**Figure 1:**
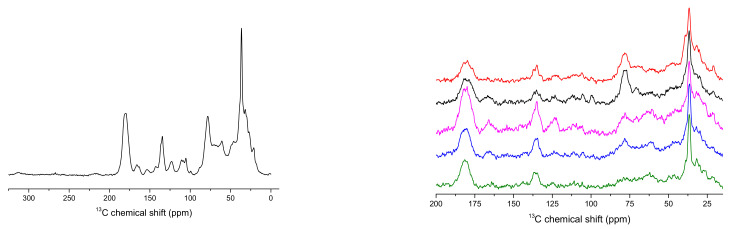
Average ^13^C CPMAS NMR spectrum of bee pollen (**left**) and spectra of the selected samples (**right**).

Bee pollen is a complex mixture that is rich in many nutrients, including carbohydrates, lipids, proteins, and amino acids. The variety of sources from which it can originate results in significant differences in relative proportions of constituents, which can be easily observed in the solid-state NMR spectra. An average ^13^C NMR bee pollen spectrum and the spectra of the selected pollens for which the most pronounced changes of relative peak intensities were observed are shown in Figure 1. Spectra collected for the complete set of analyzed samples are presented in Figure S1 in the Supplementary Materials.

### 3.1. Solid-State NMR Spectra

**Figure 2:**
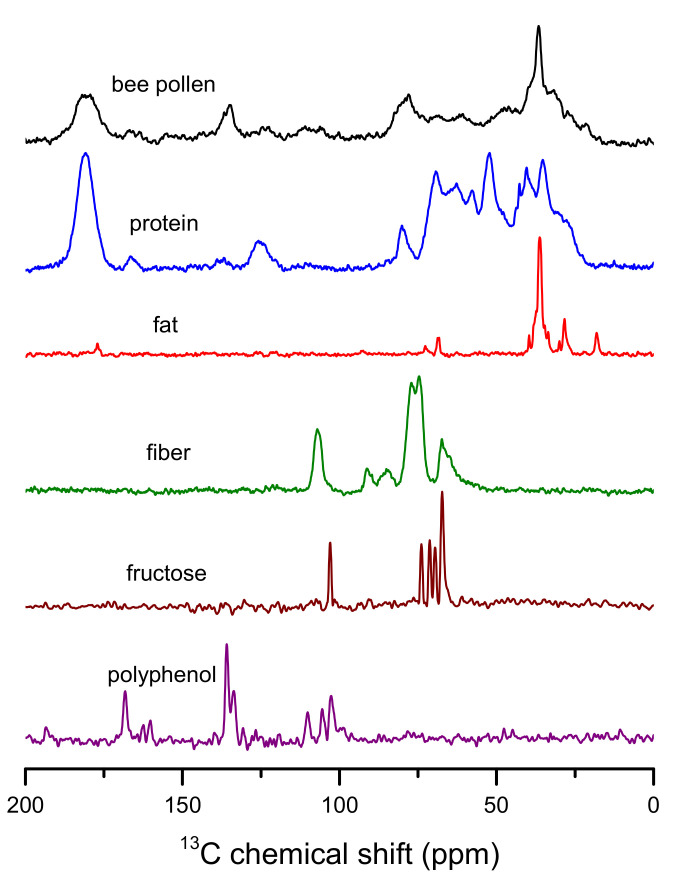
^13^C CPMAS NMR spectra of bee pollen and reference compounds. Protein: soy protein isolate; fat: coconut oil; fiber: wheat fiber; polyphenol: gallic acid.

Certain groups of nutrients and other chemical compounds can be detected in bee pollen based on its ^13^C NMR spectrum [20]. The most intense peak in the spectrum appears at about 36 ppm. This signal, together with the peak at 30 ppm, characterizes the presence of aliphatic fatty acids. Signals in the 30–40 ppm range can be attributed to –(CH_2_)_n_– fragments of lipids and phospholipids, whereas contributions at lower shifts can be assigned to methylene of the CH_2_-CH= moieties and terminal methyl groups of lipids (Figure 2). Another characteristic peak arising from the presence of fatty acid carbonyl group can be found at about 175 ppm, but this signal is overlapped by protein contribution. Chemical shifts in the 60–110 ppm range are typical for polysaccharides, which are present in large amounts in the studied product. In this range, the signal of the C1-C4 linkage from glycosidic bonds of polymeric sugars makes a significant contribution at about 78 ppm, whereas peaks in the 90–110 ppm range can be assigned to the presence of anomeric carbons. Signals of proteins, the content in the studied samples of which reaches up to 29% (*w*/*w*), give significant shares in the NMR spectrum of bee pollen. Chemical shifts for peptides’ aliphatic carbons can be observed in the range below 40 ppm, which partially overlap lipid contributions. The presence of signals of the C=O groups can be detected in the form of a broad peak at about 180 ppm, while the other protein contributions can be found in the 45–78 ppm range and at about 123 and 165 ppm. Constituents having a distinct spectral representation in ^13^C CPMAS NMR spectrum of bee pollen are polyphenolic compounds. Although their content usually does not exceed 1.5% of the product mass, the contribution of aromatic carbons can be detected in the 100–170 ppm range of the spectrum with characteristic doublet at about 135 ppm. Weak broad signals in the 140–160 ppm spectrum range can be attributed to the presence of tannins in the studied material. Solid-state NMR spectra recorded for the substances that can be found in bee pollen are shown in Figure 2.

### 3.2. Principal Component Analysis (PCA)

**Figure 3:**
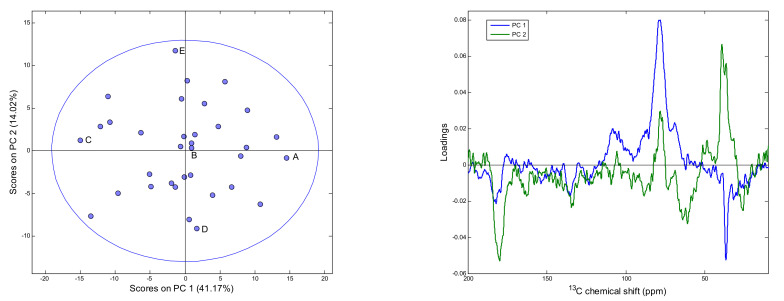
PC1/PC2 scores (**left**) and plot of the first two PCA loadings (**right**).

PCA was performed on a matrix of solid-state NMR spectra recorded for the analyzed pollen samples. The first five principal components explained about 75% of a total variance in the spectral data. In the case of higher PCs, they describe mainly variability related to the spectral noise. As is visible in Figure 1, solid-state NMR spectra of the analyzed material are characterized by a rather moderate value of the signal-to-noise (S/N) ratio. The scores plot and the loadings of the first two PCs are presented in Figure 3. In the PC1 loadings plot, the contributions related to variability of lipids and phospholipids, i.e., negative signals at 36 and 180 ppm, polysaccharides, in the form of positive signals with maxima at about 70 and 110 ppm, and polyphenols content, as negative signal at 130 ppm, can be distinguished. In the PC2 plot, the most pronounced positive signals of aliphatic carbons in the 30–40 ppm range and at 80 and 110 ppm related to polysaccharides are visible. A strong negative feature present in this plot at 170 ppm, together with features that appear at about 25, 70, 130 and 160 ppm, can be attributed to protein content variability. Comparison of ^13^C NMR spectra of bee pollen samples located in different positions of the PC1/PC2 coordinate system is presented in Figure S2 in the Supplementary Materials.

### 3.3. Construction of PLS Models

To construct quantitative models for the selected parameters, the bee pollen solid-state NMR spectra of the studied samples were combined with the results of reference analyses performed according to the standard protocols, as described in Section 2.2. PLS models were built by selecting the spectral ranges for which the highest values of calibration parameters (R and R_cv_) and the lowest RSEP error values were obtained. Optimization of ranges was supported by the plots of the variable importance in projection scores, and the number of PLS factors was determined based on the root-mean-square error of cross-validation (RMSECV) plots. Various data treatment methods were tested, but only the parameters of the most robust models are discussed.

#### 3.3.1. Nutrients Content Modeling

Bee pollen’s chemical composition and nutritional value shows considerable variability between plant species. For example, Feas et al. [2] reported bee pollen containing the following nutrients: carbohydrates (61.2–70.6%), protein (19.1–27.1%), and fat (4.3–6.3%). Campos et al. [31] reported greater variability of these compounds, i.e., carbohydrates ranged from 13 to 55%, proteins and lipids in the 10–40% and 1–13% range, respectively. A similar concentration range of the nutrients was found in Brazilian and Colombian bee pollen, with 16.1–32.1% for protein and 2.8–9.7% for fat [4,32]. In the case of mono-floral pollen, an analysis content of total sugars ranged from 34.7% (*Hedera helix*) to 63.5% (*Actinidia chinensis*). Fructose and glucose constituted 94% of the total sugars, ranging from 15.5% (*Lamium amplexicaule*) to 33.5% (*A. chinensis*) and from 13.6 (*H. helix*) to 27.7% (*A. chinensis*), respectively [33].

**Figure 4:**
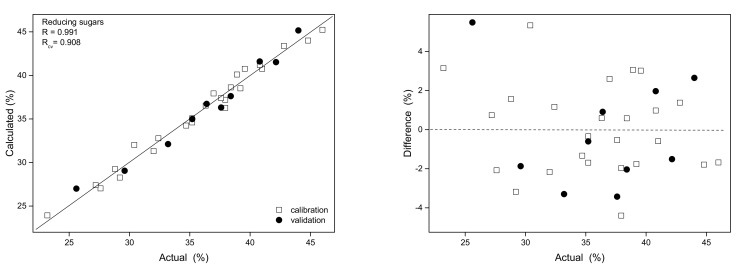
Prediction curve (**left**) and relative errors (**right**) for reducing sugars content modeling based on ^13^C NMR spectra.

In the studied pollen samples, the content of reducing sugars varied in the 23.2–46.0% range. The spectra was in the 55–220 ppm range and four factors were applied for PLS modeling. The obtained prediction curve was characterized by a correlation coefficient (R) value of 0.991. The prediction curve and plot of relative errors are shown in Figure 4, and the (RMSECV) parameter and the variable importance in projection (VIP) scores are presented in Figure S3 in the Supplementary Materials. Internal validation of the model resulted in a correlation coefficient of cross-validation (R_cv_) value of 0.908 while RSEP errors for calibration and validation sets amounted to 2.4%. The parameters of the obtained regression models are summarized in Table 2.

Models of a similar quality were obtained for the remaining nutrients present in the studied bee pollens. In our samples, protein concentration varied from 14.9 to 28.8% (*w*/*w*) and fat content changed in the 7.6–12.3% (*w*/*w*) range. PLS models based on the solid-state NMR spectra were characterized by high R (R_cv_) values. These were 0.992 (0.899) for proteins and 0.991 (0.862) for fat. Quantification of validation samples resulted in the RSEP errors of 2.2% for fat and 2.5% for protein, whereas, in the case of calibration samples, they were 1.8% and 2.0%, respectively (Table 2). The prediction curves, plots of relative errors, RMSECV parameter, and VIP scores for the studied compounds are presented in Figures S4 and S5 in the Supplementary Materials.

#### 3.3.2. Modeling of Total Polyphenols Content and Antioxidant Activity

Phenolic compounds are an important group of constituents of bee products which have a noticeable effect on biological activity and pro-health properties. Among apicultural products, bee pollen is considered as the one containing the highest amount of phytochemicals, i.e., flavonoids, anthocyanidins, catechins, phenolic acids, and triterpene compounds. Therefore, bee pollen can be used to increase polyphenols content in food products [34].

Polyphenols content in multifloral samples change typically in the 5–30 mg GAE/g range, but for some individual plant species, values exceeding 120 mg GAE/g are reported [35]. As examples, the TP content in pollen from Italy ranged between 5.78 to 20.15 mg GAE/g [36]; similar values in the 12.9–19.8 mg GAE/g range were found in pollens from Portugal [2]. In the case of bee pollen from Brazil and collected from the Sonoran Desert, the polyphenols content was found in the 6.5–29.2 and in 15.9–34.9 mg GAE/g range, respectively [37,38]. The TP content in bee pollens depends on the samples’ botanical origin, but the method of extract preparation can also influence the assay [6,35,39]. In bee pollen samples originating from the south of Poland, the TP in ethanol–water extracts was found to be 27.03 mg GAE/, while, in pepsin extracts, it resulted in 13.24 mg GAE/g [6]. Since there are many ways to express the antioxidant activity of bee pollen extracts, comparisons between different studies are not straightforward [35,40]. As an example, the ABTS antioxidant activity for multifloral pollen samples from Portugal and Spain ranged between 119 and 276.8 μM TE/g [41], while, in samples from Poland, it reached the highest value of 178 μM TE/g [6].

In the studied samples, TP content varied in the 4.2–14.6 mg GAE/g and ABTS antioxidant activity was in the 117.2–264.8 μM TE/g range. These two parameters are highly correlated (R = 0.84), but antioxidant activity not only depends on TP content, but can also be influenced by the presence of other compounds, including vitamins C and E and β-carotene. Calibration models were developed utilizing spectral ranges listed in Table S1 in the Supplementary Materials, applying 3–4 PLS factors. These models were characterized by correlation coefficient values in the 0.985–0.995 range and values of the R_cv_ in the 0.881–0.887 range; prediction curves are presented in Figures S6 and S7 in the Supplementary Materials. The RSEP error values obtained for polyphenols content and ABTS antioxidant activity determination change in the 2.7–2.8% and 3.5–3.8% range for calibration and validation sets, respectively (Table 2).

#### 3.3.3. Modeling of Nitrogen, Carbon and Sulfur Content

Studies reporting elemental analysis of bee pollen are abundant. It is recognized that the content of macroelements (K, P, S, Ca and Mg, followed by Na, Fe, Mn, Al and Zn) as well as microelements and trace elements can vary in bee pollen considerably [42,43]. On the contrary, works describing NHC analysis are scarce. Carbon content in the 46–48%, and nitrogen in the 2.2–7.4% range was found by Filipiak et al. [44].

Solid-state NMR spectra of bee pollen were combined with the results of NHCS elemental analyses. Calibration models were built for the sum of these elements and separately for each element except hydrogen. For these models, regression coefficient values changed in the 0.975–0.992 range; appropriate prediction plots are presented in Figure S8–S11 in the Supplementary Materials. Internal validation of these models resulted in slightly lower R_cv_ values when compared to the parameters of PLS models built for nutrients and polyphenols (Table 2). In the case of carbon content modeling and the sum of the NHCS values, the RSEP error values changed in the 0.3–0.5% and 0.4–0.6% ranges for calibration and validation samples, respectively. The calibration model constructed for nitrogen content determination based on the solid-state NMR spectra was characterized by the R/R_cv_ values of 0.994/0.922. The RSEP errors amounted to 1.9% for calibration and validation sets analysis. The amount of nitrogen in bee pollen samples found through elemental analysis can be used to determine protein content. Direct comparison of the RSEP values from Table 2 clearly shows similarity of the calibration parameters for protein and nitrogen content modeling. Modeling of sulfur content, which in our samples varied in the 0.06–0.24% range, resulted in the RSEP_cal_ and RSEP_val_ error values of 4.2% and 6.1%, respectively.

#### 3.3.4. Modeling of pH

Signals from the ^13^C CPMAS NMR spectra yield information about the chemical environment of the nuclei, providing structural data on a substance being analyzed. Given that a number of the compounds’ physical features depend directly on their chemical composition, determination of some additional parameters based on the NMR spectra seems feasible. In the course of our study, the pH values measured for bee pollen solutions were correlated with the spectral data. In the analyzed products, the pH varied in the 4.1–5.8 range which is in accordance with the other studies, indicating typical acidic character of the product [2,32]. The PLS model consisted of applying 7 LVs which allowed determination of this parameter with RSEP errors of 1.4% and 2.1% for calibration and validation samples (see Table 2 and Figure S12 in the Supplementary Materials).

## 4. Conclusions

The obtained results indicate that ^13^C CPMAS NMR spectroscopy can be used to directly quantify nutrients and selected physiochemical parameters of bee pollen samples in their native state. By applying multivariate modeling techniques, these analyses can be performed simultaneously. Based on the constructed PLS models, macronutrients were quantified with RSEP_val_ errors in the 2.2–2.5% range. These errors amounted to 0.4% in the case of C quantification, 1.9% for N determination, and 6.1% for S determination. Modeling of TP content, ABTS antioxidant activity, and pH by applying spectral data resulted in the RSEP_val_ values of 3.5%, 3.8%, and 2.1%, respectively.

The presented results show that, on the basis of a single solid-state NMR spectrum of a powdered bee pollen sample, various parameters of this complex natural product can be determined without the use of solvents and separation techniques.

## Figures and Tables

**Table 1:** Characteristics of the studied bee pollen samples.

No.	Origin of Sample	TP ^a^ [mg GAE/g]	AOA ^b^ ABTS [µM TE/g]	Reducing sugars [%]	Protein [%]	Fat [%]	NHCS [%]	C [%]	N [%]	S [%]	pH
1	Legnica	12.33	168.5	27.6	22.38	9.64	56.10	45.09	3.58	0.105	4.05
2	Legnica	12.08	172.6	32.0	23.56	9.78	57.30	46.02	3.77	0.076	4.64
3	Stróże	12.54	223.1	41.0	25.00	7.58	57.51	46.13	4.00	0.112	4.55
4	Stróże	9.52	157.0	32.4	20.81	8.94	56.61	45.73	3.33	0.097	4.88
5	Sulęcin	11.46	178.6	29.6	24.13	10.20	58.11	46.65	3.86	0.061	4.66
6	Łódź	8.98	168.2	35.2	20.63	8.97	56.15	45.59	3.30	0.131	4.92
7	Malbork	7.91	149.2	42.2	17.69	8.95	56.04	45.87	2.83	0.103	4.59
8	Otmuchów	9.35	181.9	35.2	22.88	9.17	57.75	46.58	3.66	0.158	4.76
9	Otmuchów	8.11	160.7	36.4	19.75	7.89	56.22	45.63	3.16	0.125	4.96
10	Stróże	4.16	117.2	38.9	15.88	7.90	53.37	43.73	2.54	0.102	4.97
11	Sokołów Małopolski	13.01	235.3	42.8	25.63	7.76	57.98	46.52	4.10	0.162	4.74
12	Wadowice	7.69	167.0	39.6	16.94	7.84	55.95	45.34	2.71	0.118	5.59
13	Uścikowo	6.11	141.6	38.4	16.88	8.42	58.36	46.50	2.70	0.111	5.29
14	Malbork	9.41	160.8	40.8	15.69	7.87	56.66	45.92	2.51	0.180	5.29
15	Suchlica	7.12	152.9	44.8	26.19	8.38	57.69	46.75	4.19	0.202	4.54
16	Łódź	10.31	209.2	28.8	25.00	8.16	56.15	47.80	4.00	0.165	5.33
17	Legnica	9.70	180.7	39.2	21.88	11.16	58.72	44.73	3.50	0.143	4.84
18	Częstochowa	8.43	140.3	46.0	20.25	9.24	59.73	46.76	3.24	0.174	5.27
19	Częstochowa	10.61	208.6	34.7	25.50	7.56	55.56	46.13	4.08	0.165	5.25
20	Wrocław	7.80	155.7	36.3	21.06	8.66	58.46	45.10	3.37	0.145	4.96
21	Stróże	8.90	186.5	37.6	21.38	8.21	57.24	46.22	3.42	0.239	5.13
22	Wola Węgierska	10.32	172.7	37.9	21.13	8.26	55.93	46.86	3.38	0.172	4.86
23	Bielsko-Biała	9.84	190.6	37.9	21.75	8.04	57.37	46.05	3.48	0.123	4.81
24	Rogóż	8.15	169.8	37.0	20.00	10.01	57.45	46.46	3.20	0.160	4.83
25	Otmuchów	5.75	119.3	33.2	16.13	8.49	57.27	44.57	2.58	0.125	4.81
26	Wambierzyce	9.61	185.7	35.2	21.13	8.53	56.98	45.81	3.38	0.136	5.50
27	Kozaki	7.32	146.4	37.6	17.94	7.98	56.06	45.48	2.87	0.161	5.40
28	Byków	9.78	165.5	30.4	19.81	9.71	56.39	45.45	3.17	0.213	4.06
29	Byków	13.39	191.2	38.4	24.69	9.85	58.83	46.93	3.95	0.191	4.41
30	Kamienna Góra	14.57	264.8	40.8	28.81	8.25	59.37	47.47	4.61	0.192	5.80
31	mix1	13.18	193.8	27.2	23.94	9.07	58.10	46.42	3.83	0.204	6.30
32	mix2	13.92	210.8	23.2	27.63	12.28	58.92	46.72	4.42	0.228	4.74
33	mix3	11.86	203.1	29.2	27.50	11.15	59.55	47.60	4.40	0.189	4.98
34	mix4	12.84	196.3	25.6	25.13	11.98	59.31	47.32	4.02	0.227	5.14
35	mix5	12.92	205.2	44.0	14.88	8.39	55.72	45.46	2.38	0.130	5.09

^a^ total polyphenolic compounds, ^b^ antioxidant activity.

**Table 2:** Details of the constructed PLS models.

Analyzed Feature	Parameter					
	RSEP_cal_	RSEP_val_	R	R_cv_	Number of LV	Pretreatment
(Range)	[%]	[%]				
Reducing sugars (23.2–46.0%)	2.37	2.38	0.991	0.908	4	MSC
Protein (14.9–28.8%)	2.02	2.52	0.992	0.899	4	SNV
Fat (7.6–12.3%)	1.79	2.22	0.991	0.862	4	SNV
sum of NHCS (53.4–59.7%)	0.47	0.62	0.975	0.766	5	none
C (44.7–47.8%)	0.32	0.42	0.981	0.780	5	none
N (2.4–4.6%)	1.85	1.89	0.994	0.922	4	SNV
S (0.06–0.24%)	4.23	6.07	0.992	0.768	5	MSC
Total polyphenols (4.2–14.6 mg GAE/g)	2.73	3.51	0.995	0.887	4	MSC
Antioxidant activity ABTS (117.2–264.8 µM TE/g)	2.80	3.83	0.985	0.881	3	none
pH (4.1–5.8)	1.43	2.14	0.994	0.777	7	none

## Data Availability

Data is contained within the article and supplementary material.

## Supplementary Materials

The following are available online at https://www.mdpi.com/article/10.3390/antiox10071123/s1, Figure S1: ^13^C CPMAS NMR spectra of the analyzed bee pollen samples, Figure S2: ^13^C NMR spectra of selected bee pollen samples, Figure S3: Modeling of reducing sugars content in pollen samples on the basis of ^13^C NMR spectra, Figure S4: Modeling of protein content in pollen samples on the basis of ^13^C NMR spectra, Figure S5: Modeling of fat content in pollen samples on the basis of ^13^C NMR spectra, Figure S6: Modeling of total polyphenolic compounds content in pollen samples on the basis of ^13^C NMR spectra, Figure S7: Modeling of ABTS antioxidant activity in pollen samples on the basis of ^13^C NMR spectra, Figure S8: Modeling of pH of pollen samples on the basis of ^13^C NMR spectra, Figure S9: Modeling of NHCS content in pollen samples on the basis of ^13^C NMR spectra, Figure S10: Modeling of C content in pollen samples on the basis of ^13^C NMR spectra, Figure S11: Modeling of N content in pollen samples on the basis of ^13^C NMR spectra, Figure S12: Modeling of S content in pollen samples on the basis of ^13^C NMR spectra, Table S1: Spectral regions used for PLS modeling.

## References

[1] Denisow B., Denisow-Pietrzyk M. (2016). Biological and therapeutic properties of bee pollen: A review. J. Sci. Food Agric..

[2] Feas X., Vazquez-Tato M.P., Estevinho L., Seijas J.A., Iglesias A. (2012). Organic bee pollen: Botanical origin, nutritional value, bioactive compounds, antioxidant activity and microbiological quality. Molecules.

[3] Thakur M., Nanda V. (2020). Composition and functionality of bee pollen: A review. Trends Food Sci. Technol..

[4] Almeida-Muradian L.B., Pamplona L.C., Coimbra S., Barth O.M. (2005). Chemical composition and botanical evaluation of dried bee pollen pellets. J. Food. Comp. Anal..

[5] Leja M., Mareczek A., Wyzgolik G., Klepacz-Baniak J., Czekonska K. (2007). Antioxidative properties of bee pollen in selected plant species. Food Chem..

[6] Rzepecka-Stojko A., Stojko J., Kurek-Gorecka A., Gorecki M., Kabala-Dzik A., Kubina R., Mozdzierz A., Buszman E. (2015). Polyphenols from bee pollen: Structure, absorption, metabolism and biological activity. Molecules.

[7] Sattler J.A.G., de Melo I.L.P., Granato D., Araujo E., de Freitas A.D., Barth O.M., Sattler A., de Almeida-Muradian L.B. (2015). Impact of origin on bioactive compounds and nutritional composition of bee pollen from southern Brazil: A screening study. Food Res. Int..

[8] Cornara L., Biagi M., Xiao J.B., Burlando B. (2017). Therapeutic properties of bioactive compounds from different honeybee products. Front. Pharmacol..

[9] Hatzakis E. (2019). Nuclear Magnetic Resonance (NMR) Spectroscopy in Food Science: A Comprehensive Review. Compr. Rev. Food Sci. F..

[10] Bertocchi F., Paci M. (2008). Applications of high-resolution solid-state NMR spectroscopy in food science. J. Agric. Food. Chem..

[11] Parlak Y., Guzeler N. (2016). Nuclear magnetic resonance spectroscopy applications in foods. Curr. Res. Nutr. Food Sci..

[12] Ogrinc N., Kosir I.J., Spangenberg J.E., Kidric J. (2003). The application of NMR and MS methods for detection of adulteration of wine, fruit juices, and olive oil. A review. Anal. Bioanal. Chem..

[13] Santos C.M.M., Silva A.M.S., Cardoso S.M., Silva A.M.S. (2016). Valuable analytical tools in analysis of honeybee plant-derived compounds: Nuclear magnetic resonance spectroscopy. Chemistry, Biology and Potential Applications of Honeybee Plant Derived Products.

[14] Siddiqui A.J., Musharraf S.G., Choudhary M.I., Rahman A.U. (2017). Application of analytical methods in authentication and adulteration of honey. Food Chem..

[15] Bertelli D., Lolli M., Papotti G., Bortolotti L., Serra G., Plessi M. (2010). Detection of honey adulteration by sugar syrups using one-dimensional and two-dimensional high-resolution nuclear magnetic resonance. J. Agric. Food. Chem..

[16] Zielinski L., Deja S., Jasicka-Misiak I., Kafarski P. (2014). Chemometrics as a tool of origin determination of Polish monofloral and multifloral honeys. J. Agric. Food. Chem..

[17] Kazalaki A., Misiak M., Spyros A., Dais P. (2015). Identification and quantitative determination of carbohydrate molecules in Greek honey by employing ^13^C NMR spectroscopy. Anal. Methods.

[18] Mazzoni V., Bradesi P., Tomi F., Casanova J. (1997). Direct qualitative and quantitative analysis of carbohydrate mixtures using ^13^C NMR spectroscopy: Application to honey. Magn. Reson. Chem..

[19] Kameda T., Tamada Y. (2009). Variable-temperature ^13^C solid-state NMR study of the molecular structure of honeybee wax and silk. Int. J. Biol. Macromol..

[20] Paradowska K., Zielinska A., Kuras M., Wawer I. (2017). The composition of bee pollen color fractions evaluated by solid-state ^1^H and ^13^C NMR: Their macroelement content and antioxidant properties. J. Apic. Res..

[21] Weglinska M., Szostak R., Kita A., Nems A., Mazurek S. (2020). Determination of nutritional parameters of bee pollen by Raman and infrared spectroscopy. Talanta.

[22] Winning H., Larsen F.H., Bro R., Engelsen S.B. (2008). Quantitative analysis of NMR spectra with chemometrics. J. Magn. Reson..

[23] Hauksson J.B., Edlund U., Trygg J. (2001). NMR processing techniques based on multivariate data analysis and orthogonal signal correction. 13C CP/MAS NMR spectroscopic characterization of softwood kraft pulp. Magn. Reson. Chem..

[24] Engelsen S.B., Savorani F., Rasmussen M.A. (2013). Chemometric exploration of quantitative NMR data. eMagRes.

[25] Smolinska A., Blanchet L., Buydens L.M.C., Wijmenga S.S. (2012). NMR and pattern recognition methods in metabolomics: From data acquisition to biomarker discovery: A review. Anal. Chim. Acta.

[26] Breton R.C., Reynolds W.F. (2013). Using NMR to identify and characterize natural products. Nat. Prod. Rep..

[27] AOAC (1995). (968.28) Sugars/Total Reducing Sugars. Titration Method. Official Methods of Analysis of AOAC International.

[28] AOAC (1995). (981.10) Nitrogen Content. Kjeldahl Method. Official Methods of Analysis of AOAC International.

[29] AOAC (1995). (936.15) Fat Content in Foods. Soxhlet Extraction Method. Official Methods of Analysis of AOAC International.

[30] Re R., Pellegrini N., Proteggente A., Pannala A., Yang M., Rice-Evans C. (1999). Antioxidant activity applying an improved ABTS radical cation decolorization assay. Free Radical Biol. Med..

[31] Campos M.G.R., Bogdanov S., de Almeida-Muradian L.B., Szczesna T., Mancebo Y., Frigerio C., Ferreira F. (2008). Pollen composition and standardisation of analytical methods. J. Apic. Res..

[32] Fuenmayor C., Zuluaga C., Diaz C., Quiczan D., Cosio M., Mannino S. (2014). Evaluation of the physicochemical and functional properties of Colombian bee pollen. Rev. MVZ Cordoba.

[33] Liolios V., Tananaki C., Dimou M., Kanelis D., Rodopoulou M.A., Thrasyvoulou A. (2018). Exploring the sugar profile of unifloral bee pollen using high performance liquid chromatography. J. Food Nutri. Res..

[34] Habryka C., Socha R., Juszczak L. (2021). Effect of bee pollen addition on the polyphenol content, antioxidant activity, and quality parameters of honey. Antioxidants.

[35] Martinello M., Mutinelli F. (2021). Antioxidant Activity in Bee Products: A Review. Antioxidants.

[36] Barbieri D., Gabriele M., Summa M., Colosimo R., Leonardi D., Domenici V., Pucci L. (2020). Antioxidant, nutraceutical properties, and fluorescence spectral profiles of bee pollen samples from different botanical origins. Antioxidants.

[37] De-Melo A.A.M., Estevinho L.M., Moreira M.M., Delerue-Matos C., de Freitas A.D., Barth O.M., de Almeida-Muradian L.B. (2018). A multivariate approach based on physicochemical parameters and biological potential for the botanical and geographical discrimination of Brazilian bee pollen. Food Biosc..

[38] LeBlanc B.W., Davis O.K., Boue S., DeLucca A., Deeby T. (2009). Antioxidant activity of Sonoran Desert bee pollen. Food Chem..

[39] Oroian M., Ursachi F., Dranca F. (2020). Ultrasound-assisted extraction of polyphenols from crude pollen. Antioxidants.

[40] Kocot J., Kielczykowska M., Luchowska-Kocot D., Kurzepa J., Musik I. (2018). Antioxidant potential of propolis, bee pollen, and royal jelly: Possible medical application. Oxid. Med. Cell. Longev..

[41] Pascoal A., Rodrigues S., Teixeira A., Feas X., Estevinho L.M. (2014). Biological activities of commercial bee pollens: Antimicrobial, antimutagenic, antioxidant and anti-inflammatory. Food Chem. Toxicol..

[42] Pohl P., Dzimitrowicz A., Greda K., Jamroz P., Lesniewicz A., Szymczycha-Madeja A., Welna M. (2020). Element analysis of bee-collected pollen and bee bread by atomic and mass spectrometry—Methodological development in addition to environmental and nutritional aspects. TrAC Trends Anal. Chem..

[43] Matuszewska E., Klupczynska A., Maciołek K., Kokot Z.J., Matysiak J. (2021). Multielemental analysis of bee pollen, propolis, and royal jelly collected in west-central Poland. Molecules.

[44] Filipiak M., Kuszewska K., Asselman M., Denisow B., Stawiarz E., Woyciechowski M., Weiner J. (2017). Ecological stoichiometry of the honeybee: Pollen diversity and adequate species composition are needed to mitigate limitations imposed on the growth and development of bees by pollen quality. PLoS ONE.

