# Supplementary material for: Modeling of Antioxidant Activity, Polyphenols and Macronutrients Content of Bee Pollen Applying Solid-State 13C NMR Spectra
Source: Antioxidants (Basel). 2021 Jul 14;10(7):1123. doi: 10.3390/antiox10071123 (PMC8301116; doi:10.3390/antiox10071123)
Supplement: Supplementary file 1 [file antioxidants-10-01123-s001.zip › antioxidants-1300937-supplementary.pdf]

antioxidants

Supplementary Materials: Modeling of Antioxidant Activity, Polyphenols and Macronutrients Content of Bee Pollen Applying Solid-State ^{13}C NMR Spectra

Sylwester Mazurek [1,*], Roman Szostak [1,*], Mateusz Kondratowicz [1], Magdalena Węglińska [1], Agnieszka Kita [2] and Agnieszka Nemś [2]

[1] Department of Chemistry, University of Wrocław, 14 F. Joliot-Curie, 50-383 Wrocław, Poland; mateusz.kondratowicz@chem.uni.wroc.pl (M.K.); magdalena.weglinska@chem.uni.wroc.pl (M.W.)
[2] Department of Food Storage and Technology, Faculty of Biotechnology and Food Science, Wrocław University of Environmental and Life Sciences, 37 Chełmońskiego, 51-630 Wrocław, Poland; agnieszka.kita@upwr.edu.pl (A.K.); agnieszka.nems@upwr.edu.pl (A.N.)
* Correspondence: sylwester.mazurek@chem.uni.wroc.pl (S.M.); roman.szostak@chem.uni.wroc.pl (R.S.); Tel.: +48-71-3757-307 (S.M.); Fax.: +48-71-3757-420 (S.M.)

Table S1. Spectral regions used for PLS modeling.

**Analyzed parameter**	**Region (ppm)**
Reducing sugars	50-200
Protein	10-22 and 53-170
Fat	40-110 and 140-170
Total polyphenols	100-170
Antioxidant activity ABTS	73-158
NHCS	10-200
C	20-200
N	10-22 and 53-170
S	130-160 and 180-200
pH	110-130 and 160-180

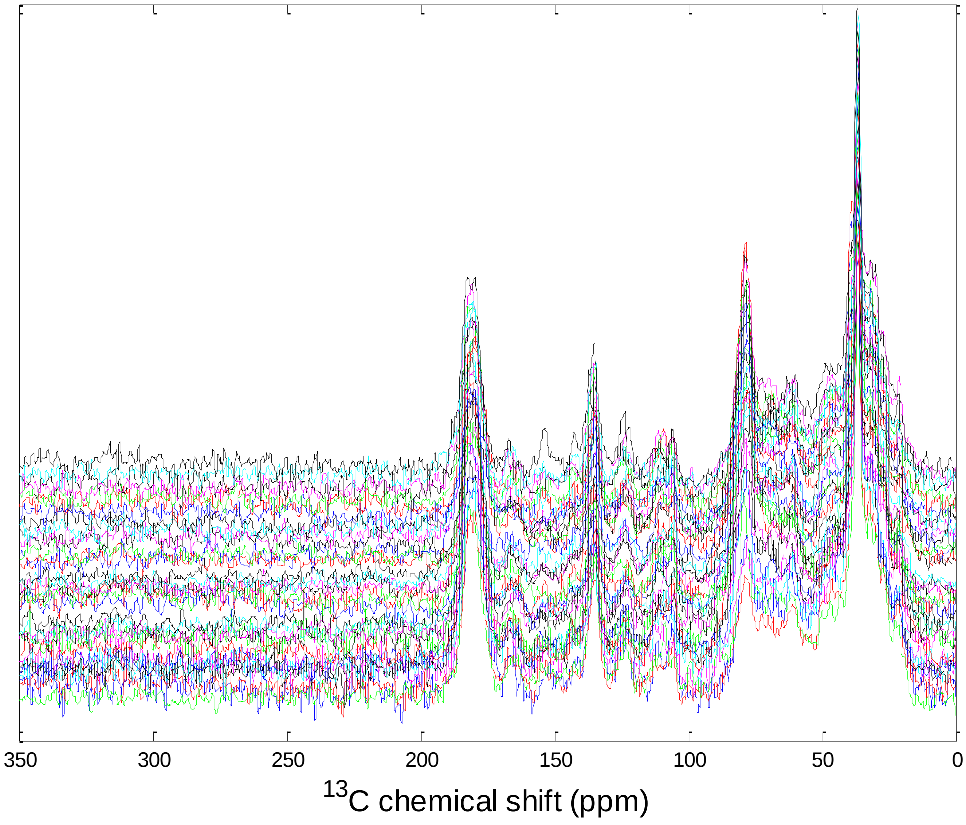

Figure S1. ^{13}C CPMAS NMR spectra of the analyzed bee pollen samples.

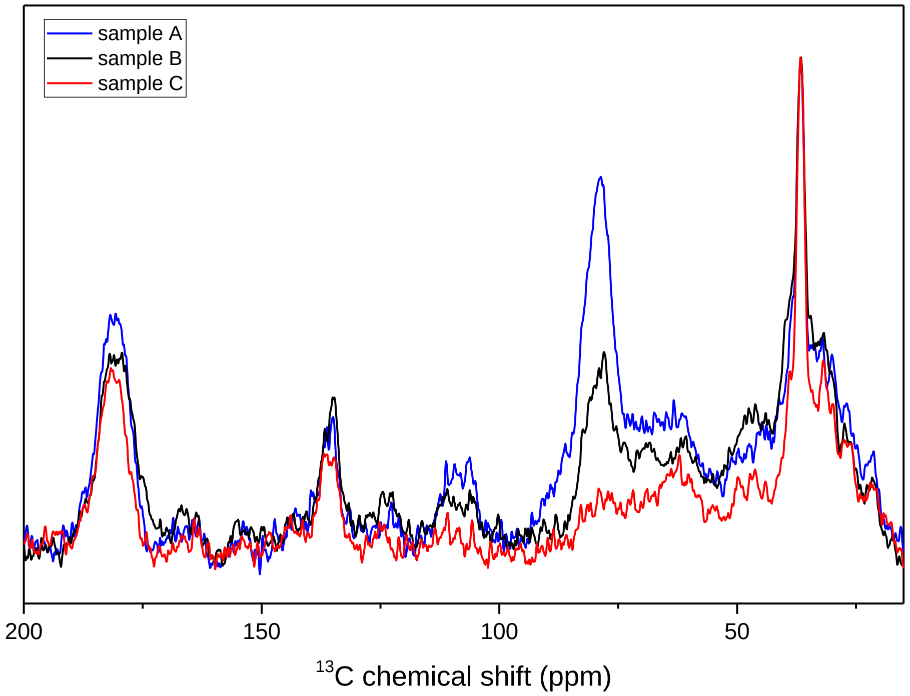

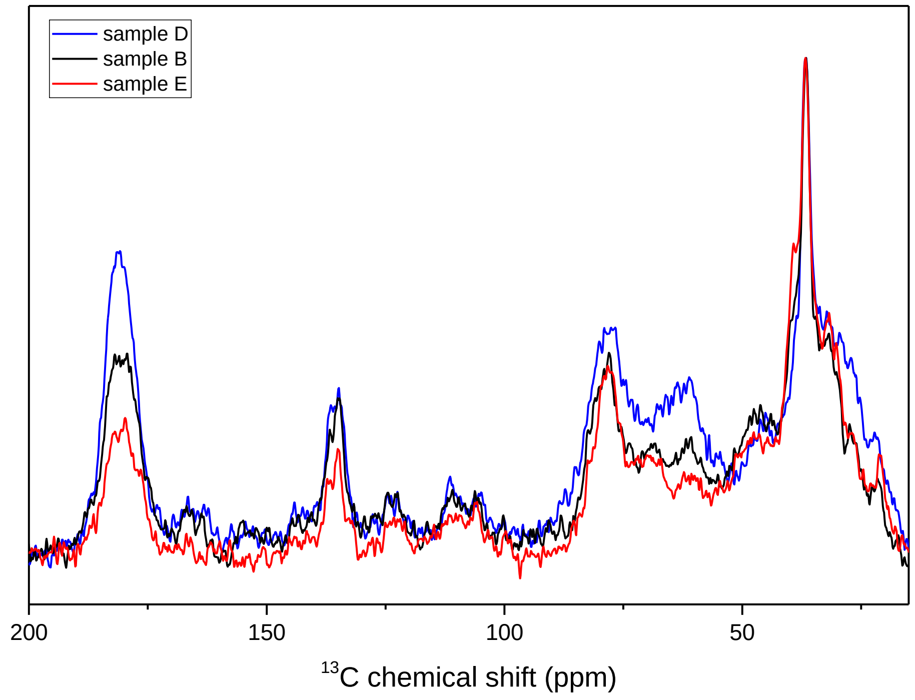

Figure S2. ^{13}C NMR spectra of selected bee pollen samples A-E; labeling as in Fig. 3.

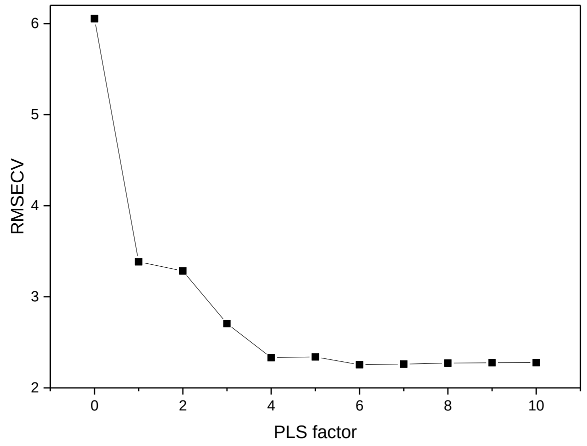

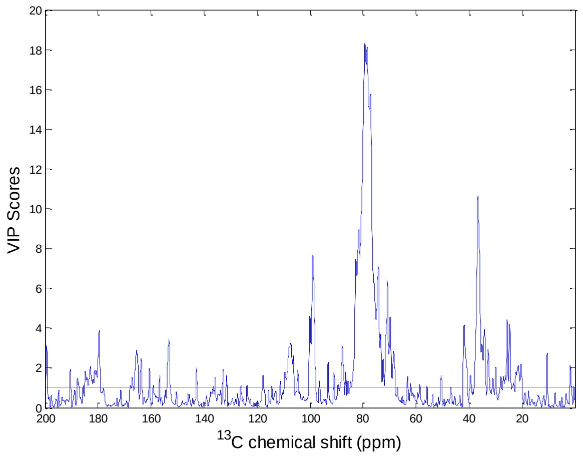

Figure S3. Modeling of reducing sugars content in pollen samples on the basis of ^{13}C NMR spectra; the RMSECV (left) and VIP scores (right) plots.

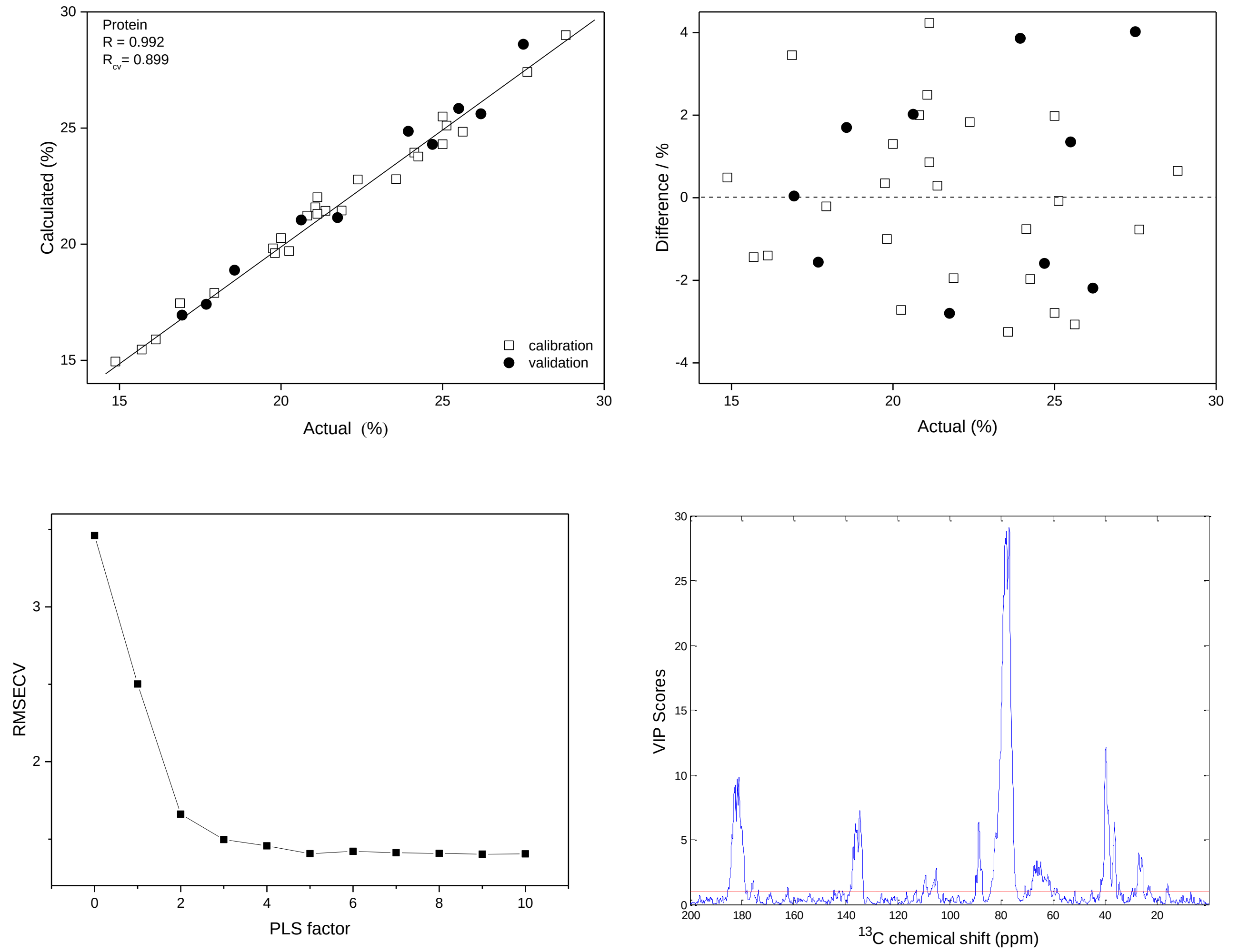

Figure S4. Modeling of protein content in pollen samples on the basis of ^{13}C NMR spectra; top panel: prediction plot (left) and relative errors (right), bottom panel: the RMSECV (left) and VIP scores (right) plots.

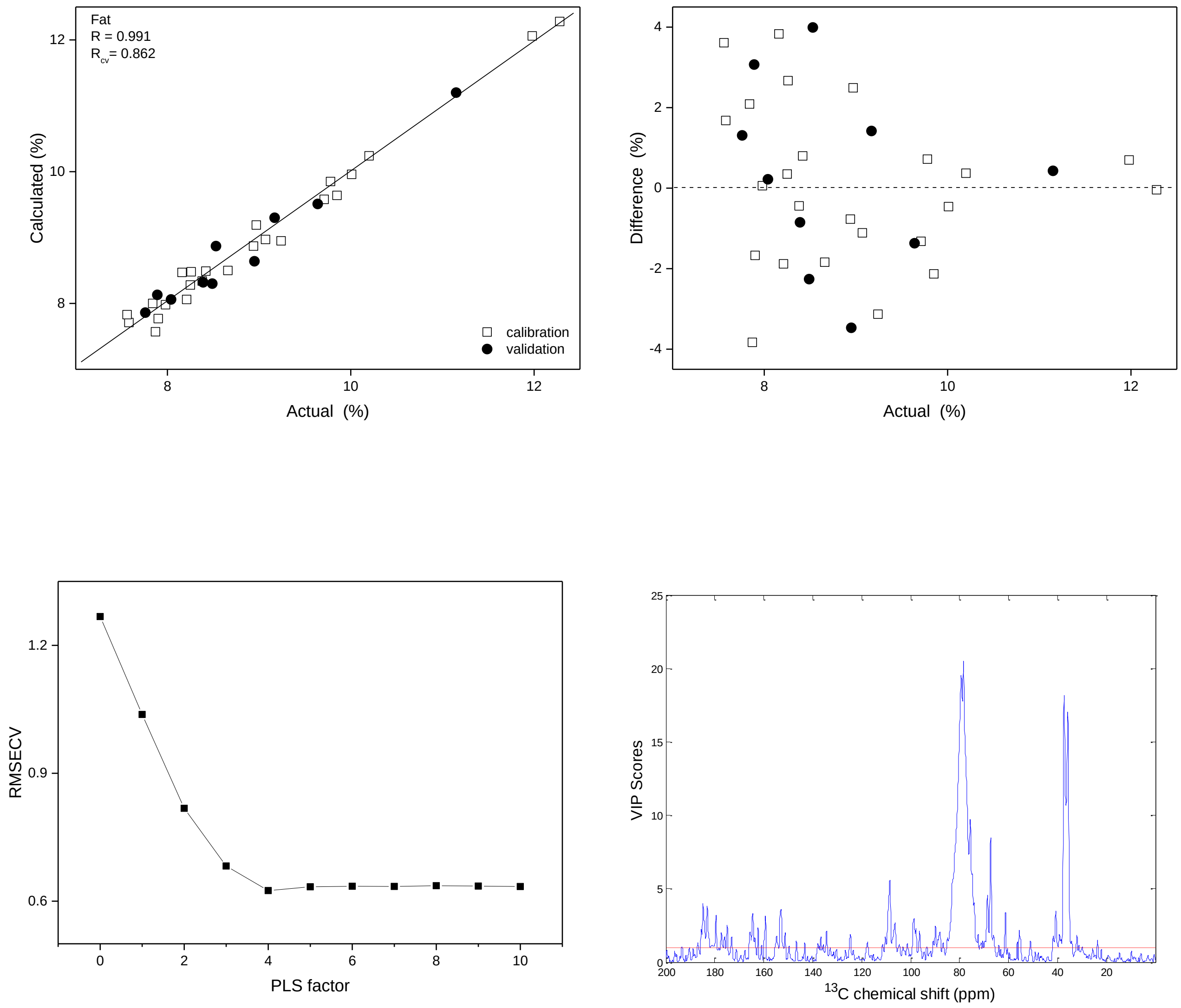

Figure S5 Modeling of fat content in pollen samples on the basis of ^{13}C NMR spectra; top panel: prediction plot (left) and relative errors (right), bottom panel: the RMSECV (left) and VIP scores (right) plots

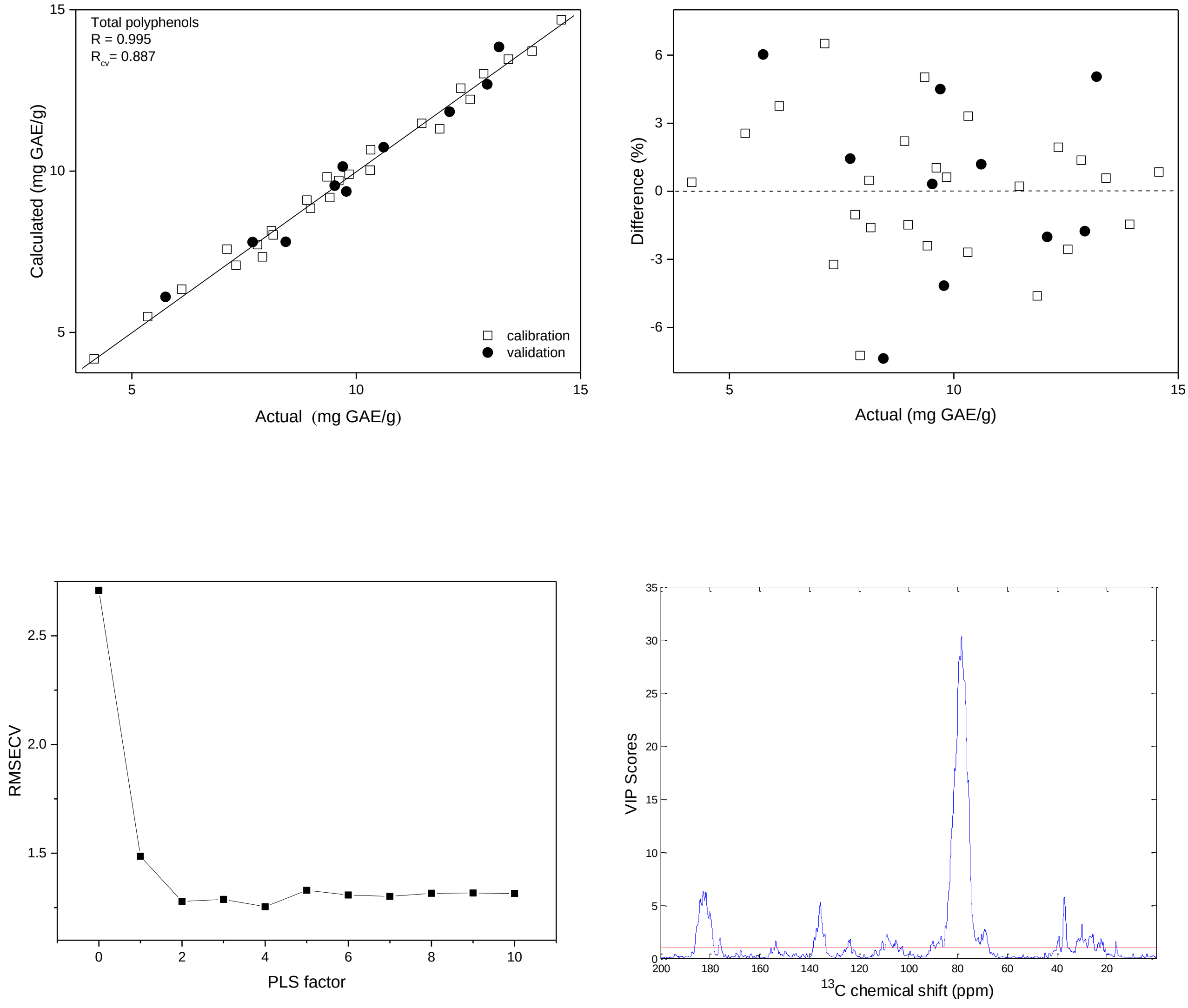

Figure S6 Modeling of total polyphenolic compounds content in pollen samples on the basis of ^{13}C NMR spectra; top panel: prediction plot (left) and relative errors (right), bottom panel: the RMSECV (left) and VIP scores (right) plots

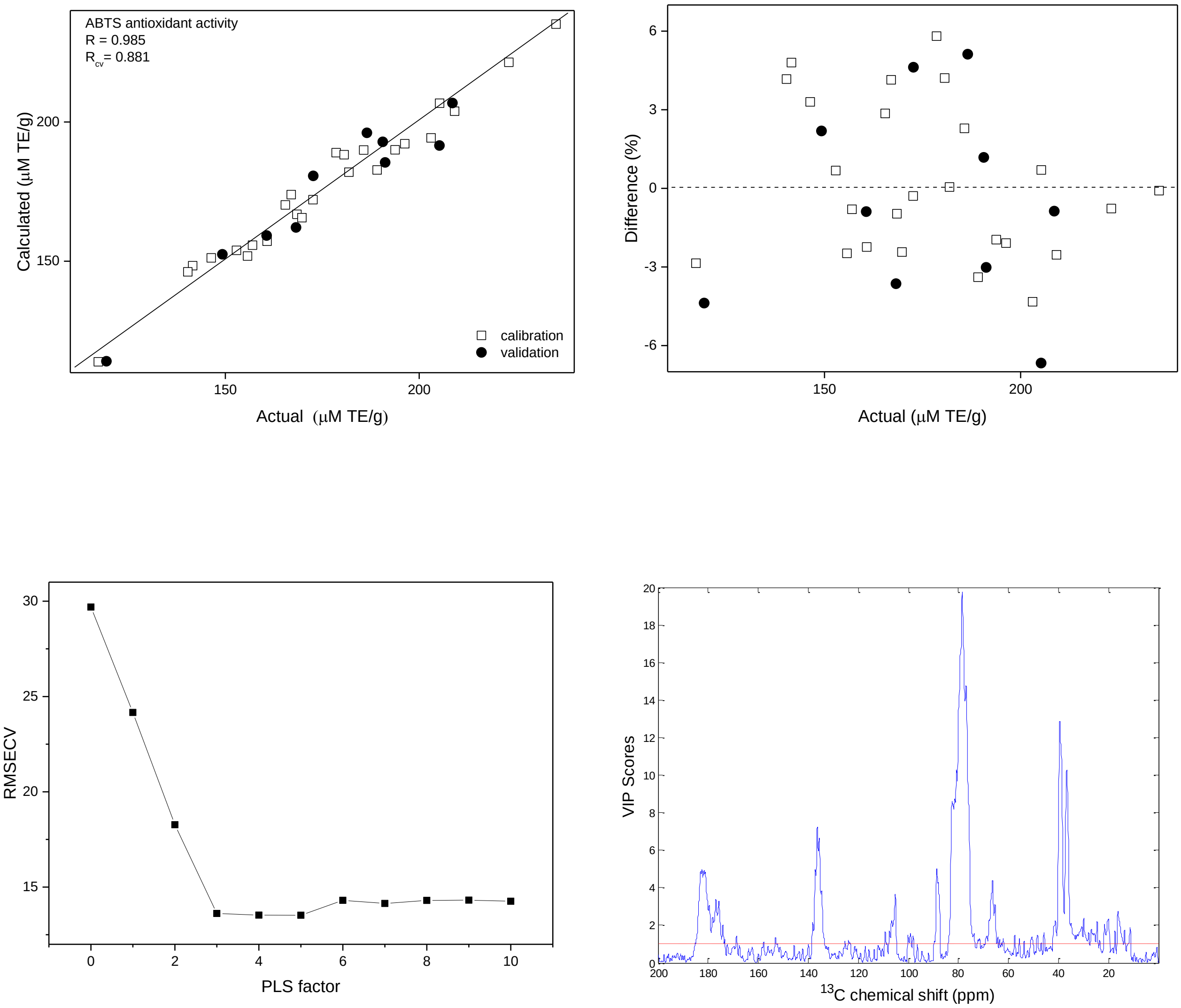

Figure S7 Modeling of ABTS antioxidant activity in pollen samples on the basis of ^{13}C NMR spectra; top panel: prediction plot (left) and relative errors (right), bottom panel: the RMSECV (left) and VIP scores (right) plots

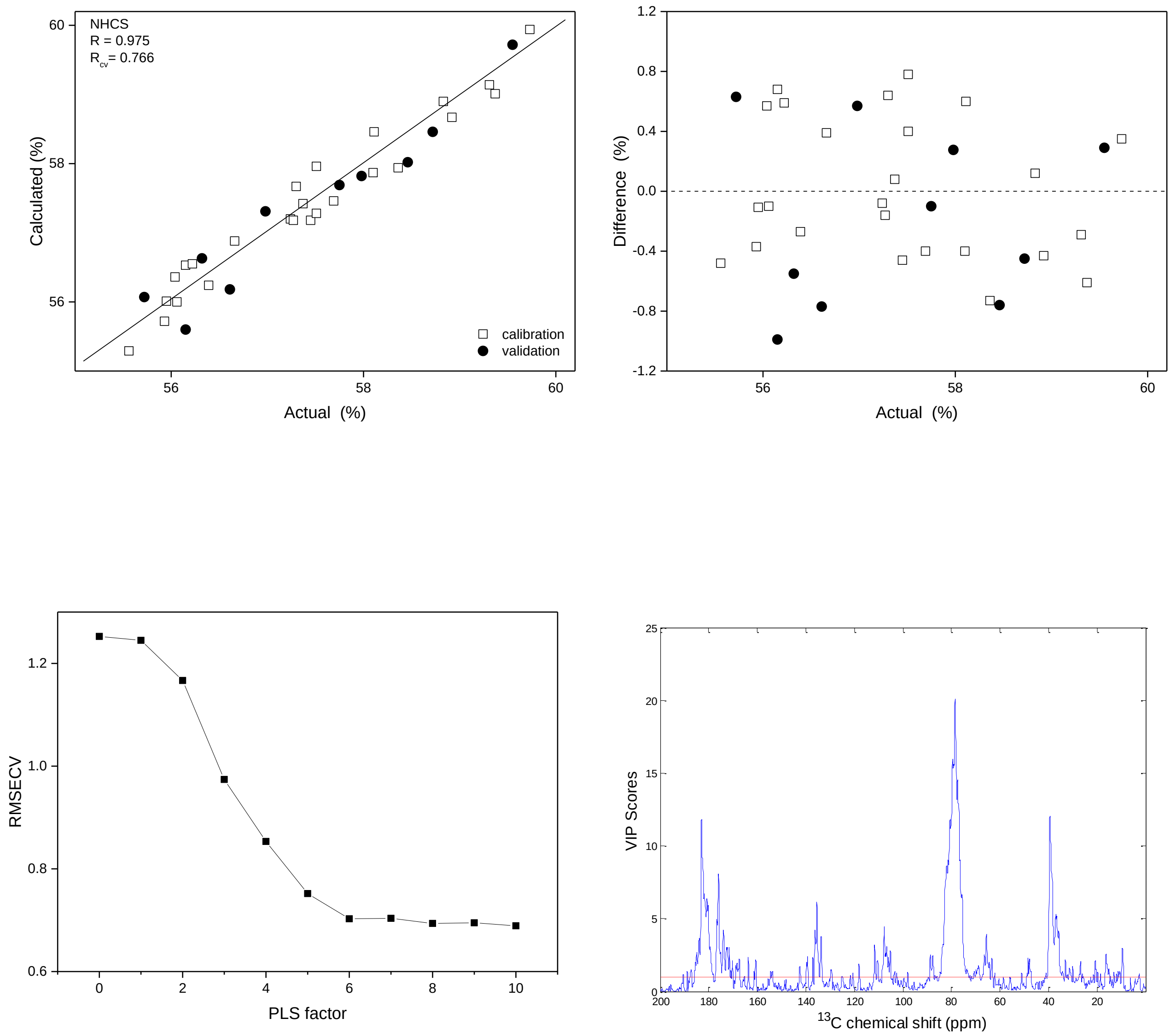

Figure S8 Modeling of NHCS content in pollen samples on the basis of ^{13}C NMR spectra; top panel: prediction plot (left) and relative errors (right), bottom panel: the RMSECV (left) and VIP scores (right) plots

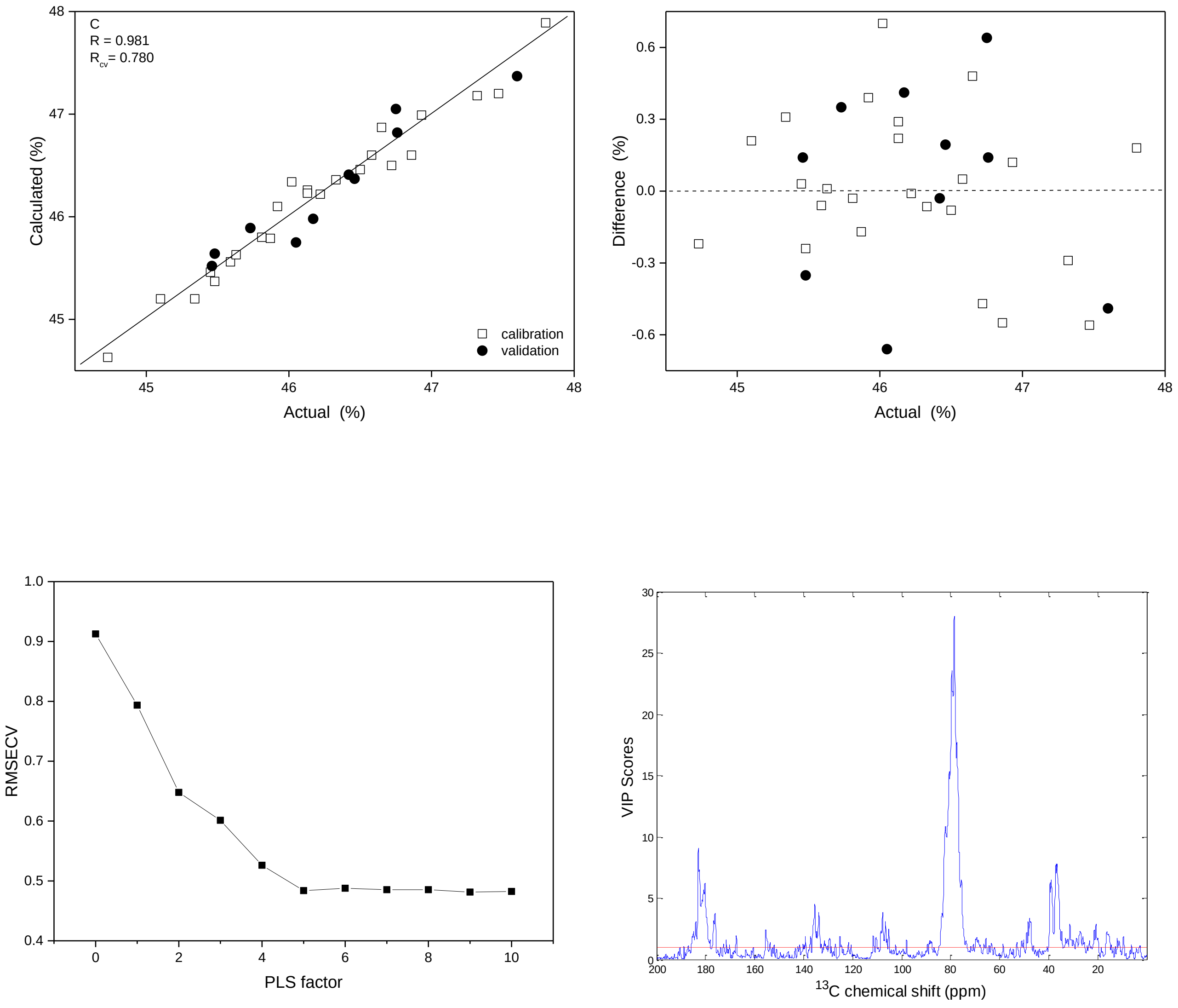

Figure S9 Modeling of C content in pollen samples on the basis of ^{13}C NMR spectra; top panel: prediction plot (left) and relative errors (right), bottom panel: the RMSECV (left) and VIP scores (right) plots

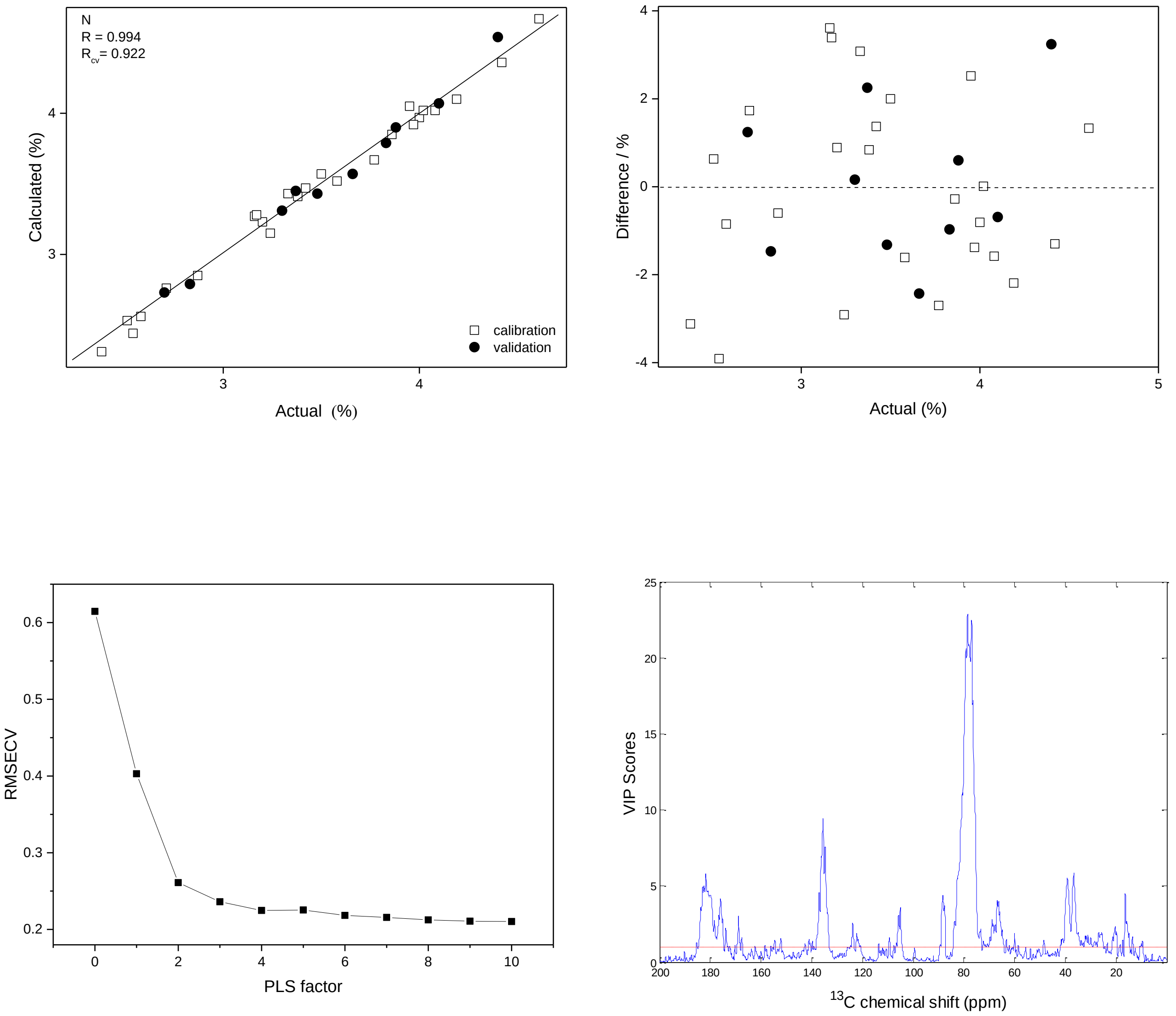

Figure S10 Modeling of N content in pollen samples on the basis of ^{13}C NMR spectra; top panel: prediction plot (left) and relative errors (right), bottom panel: the RMSECV (left) and VIP scores (right) plots

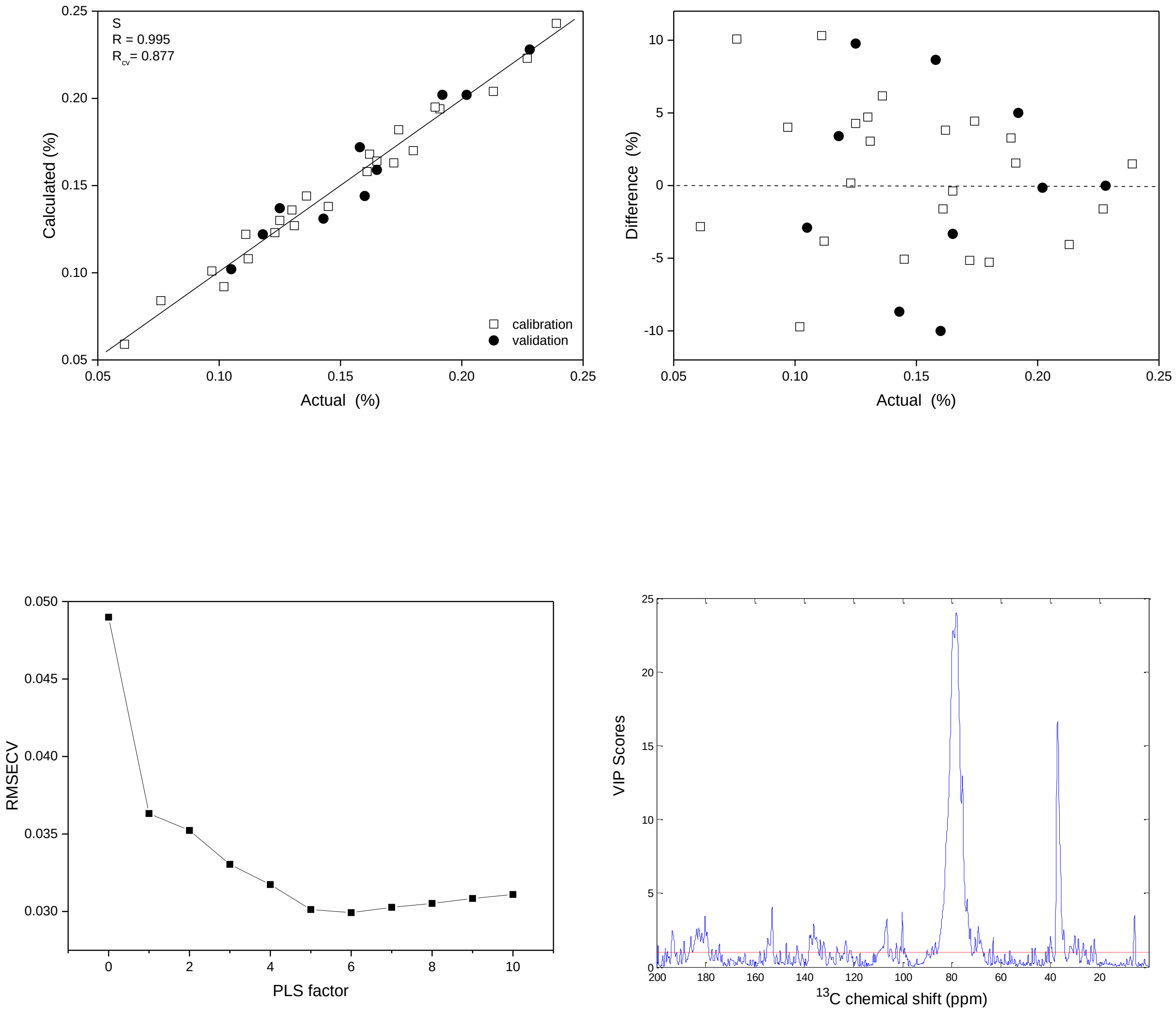

Figure S11 Modeling of S content in pollen samples on the basis of ^{13}C NMR spectra; top panel: prediction plot (left) and relative errors (right), bottom panel: the RMSECV (left) and VIP scores (right) plots

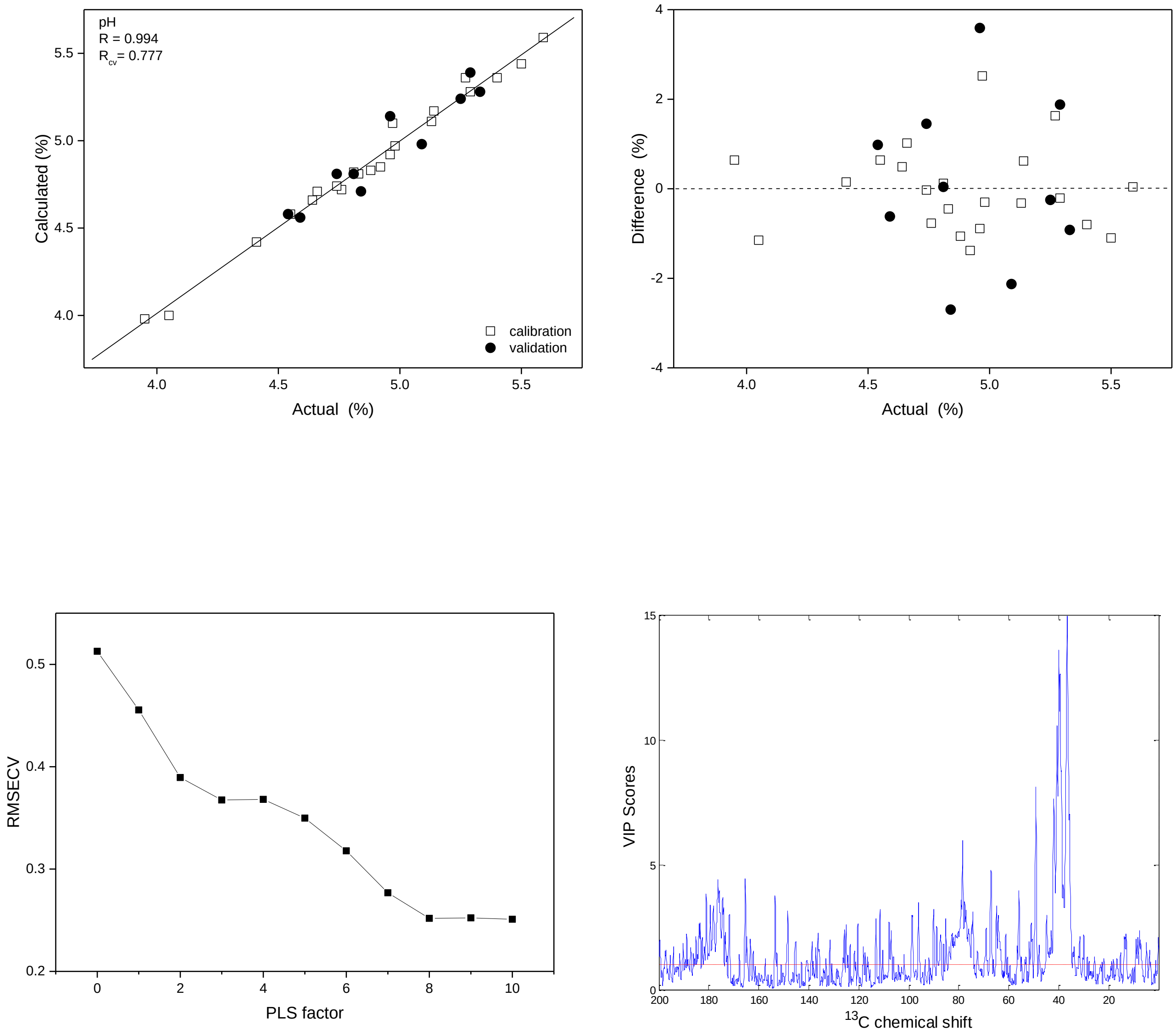

Figure S12 Modeling of pH of pollen samples on the basis of ^{13}C NMR spectra; top panel: prediction plot (left) and relative errors (right), bottom panel: the RMSECV (left) and VIP scores (right) plots
